# A case of complete splenic infarction after laparoscopic spleen-preserving distal pancreatectomy

**DOI:** 10.1186/s12893-018-0353-z

**Published:** 2018-04-10

**Authors:** Kenjiro Kimura, Go Ohira, Ryosuke Amano, Sadaaki Yamazoe, Ryota Tanaka, Jun Tauchi, Masaichi Ohira

**Affiliations:** Department of Surgical Oncology, Osaka City University School of Medicine, 1-4-3 Asahi-machi, Abeno-ku, Osaka, 545-8585 Japan

**Keywords:** Laparoscopic spleen-preserving distal pancreatectomy, Splenic infarction, Kimura’s method

## Abstract

**Background:**

Laparoscopic spleen-preserving distal pancreatectomy (LSPDP), a newly developed operative procedure, is indicated for benign and low-grade malignant disease of the pancreas. However, few studies have reported on postoperative splenic infarction after LSPDP.

**Case presentation:**

We report a case of complete splenic infarction and obliteration of the splenic artery and vein after LSPDP. The patient was a 69-year-old woman with a 35-mm cystic tumor of the pancreatic body who underwent LSPDP. Although the operation was completed with preservation of the splenic artery and vein, postoperative splenic infarction was revealed with left back pain and fluid collection around the stump of the pancreas on postoperative day 9. Fortunately, clinical symptoms disappeared within days and additional splenectomy was not needed. Splenic infarction was attributed to scattered micro-embolizations within the spleen after drawing strongly on the tape encircling the splenic vessels.

**Conclusion:**

Preserving splenic vessels in LSPDP is a demanding procedure. To prevent splenic infarction in LSPDP, we should carefully isolate the pancreatic parenchyma from the splenic vessels, and must avoid drawing tightly on the vessel loop encircling splenic vessels.

## Background

Laparoscopic spleen-preserving distal pancreatectomy (LSPDP), a newly developed operative procedure, is indicated for benign and low-grade malignant disease of the pancreas. Preservation of the spleen is preferred over routine combined splenectomy not only to avoid the risk of postoperative infectious complications after splenectomy, but also to provide longer survival with malignancy [1, 2].

LSPDP can be carried out with either preservation of the splenic vessels, as Kimura’s method [3], or with division of these vessels, as Warshaw’s method [4]. Kimura et al. provided the first report of splenic vessel-conserving spleen-preserving distal pancreatectomy for benign tumor [3]. Very few studies have evaluated outcomes for conserved splenic vessels and spleen [5–7]. Hwang et al. recently reported that 17.2% of SPDP patients showed obliteration of the splenic vein and 13.8% eventually developed collateral venous circulation around the gastric fundus and spleen [8]. Partial splenic infarction was reported in their previous reports. However, no cases of complete spleen infarction have been described.

In our institute, LSPDP using Kimura’s method was performed for 10 cases of benign pancreatic tumor. We encountered one case of complete splenic infarction and obliteration of both the splenic artery and vein. This report offers the first description of complete splenic infarction after LSPDP using Kimura’s method.

## Case presentation

**Fig. 1:**
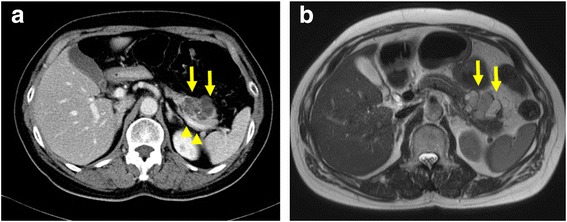
Findings from preoperative CT and MRI. CT and MRI show a cystic neoplasm (*arrow*) in the pancreatic body. Cyst diameter is 35 mm. **a** CT. **b** T2-weighted MRI. Splenic vein was normal status (*arrowhead*)

A pancreatic cystic lesion was incidentally detected in a 69-year-old woman during a clinical survey. She had no symptoms and her past history was unremarkable. Computed tomography (CT) and magnetic resonance imaging (MRI) revealed a cystic lesion with solid component in the pancreatic body (Fig. 1). Cyst diameter was 35 mm. Endoscopic ultrasonography showed this cystic lesion as a polycystic lesion with a 5-mm mural nodule. Serous cystadenoma was considered the most likely preoperative diagnosis, with a small possibility of MCN or serous cystadenocarcinoma. Because the size was beyond 3-cm and it included solid component, we decided it to be operative indication. However the risk of malignant disease was considered low, we planned to perform LSPDP using Kimura’s method.

Surgery was performed supine, 30° reverse Trendelenburg position with left-side-up adjustment. After creation of carbon dioxide pneumoperitoneum via a 12-mm umbilical port, 4 additional trocars were inserted.

After trocar placement, the greater omentum was divided using ultrasonic shears (Harmonic scalpel®; Ethicon, Cincinnati, OH) from the middle to the spleen. With the stomach elevated, the pancreatic tumor was visible from the pancreatic body to the tail. The retroperitoneum was opened along the inferior pancreatic border and further dissection was performed on the avascular plane posterior to the pancreas until the splenic vein and artery were identified. The splenic vein and artery were encircled and taped at the right side of the cystic tumor of the pancreas. When the pancreatic parenchyma was isolated from the splenic vessels, small branches from the splenic vessels were divided using ultrasonic shears and polymer ligation clips (Hem-o-lok®, Teleflex, Research Triangle Park, NC). VIO soft-coagulation system (VIO 300D; ERBE Elektromedizin, Tübingen, Germany) was used for hemostasis against oozing. Pancreatic transection was achieved using the Endo GIA™ Reinforced Reload with Tri-Staple™ Technology (Medtronic, Minneapolis, MN) with sufficient surgical margins. As the cystic tumor was tightly adherent to splenic vessels, the encircled tape was pulled to separate the tumor from the splenic vessels.

**Fig. 2:**
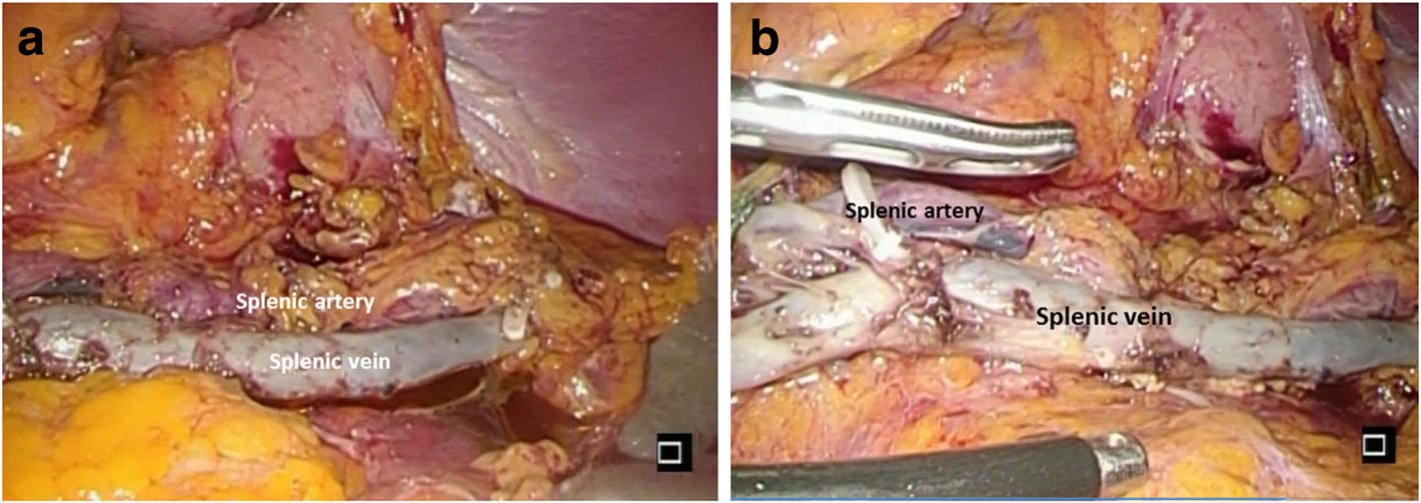
Postoperative imaging. Imaging after removal of the specimen. The splenic artery and vein are preserved along with the spleen, and they are indicated by panel labels in the Fig. 2a and b

The splenic artery and vein were preserved without dissection of the short gastric artery and vein, including left gastroepiploic vessels. The spleen was successfully conserved. The surgical specimen was retrieved in a vinyl bag and extracted through a small incision created by extending the umbilical port site. Operation time was 304 min and total blood loss was 80 g. Although the operation time was relatively long, no particular difficulties were encountered during the operation (Fig. 2).

**Fig. 3:**
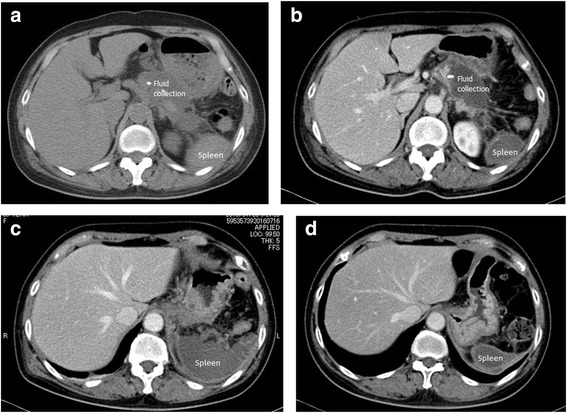
CT findings after surgery. **a** Fluid collection is evident around stump of the pancreas on postoperative day9. **b**, **c** Contrast-enhanced CT on postoperative day 15 revealed that the splenic artery and vein have been obliterated and complete splenic infarction is apparent. **d** Contrast-enhanced CT on 3 months post-operatively showed that the spleen has become atrophic and organized

For several days after the operation, the patient showed a slight fever, but abdominal pain appeared to be within the acceptable range. Concentration of amylase in the drain discharge fluid was 2225 IU/L on postoperative day 1 and 170 IU/L on postoperative day 3. The drainage tube was removed on postoperative day 5. On postoperative day 9, the patient reported left back pain and abdominal CT revealed fluid collection around the stump of the pancreas (Fig. 3a). Because postoperative pancreatic fistula was considered the most likely cause, antibiotics and octreotide were administered and eating was stopped. Clinical symptoms disappeared within a few days after this specific treatment. Contrast-enhanced CT on postoperative day 15 demonstrated obliteration of the splenic artery and vein and complete splenic infarction (Fig. 3b and c). Clinical course subsequently progressed satisfactorily. Eating was restarted on postoperative day 15, and the patient was discharged from hospital on postoperative day 25. White blood cell count was 10,100, 16,500, and 8600 /mm^3^, respectively on postoperative day 1, 3, and 6. After postoperative day 6, white blood cell count was within normal range. Infectious symptom was not obvious in the clinical course. Because we recognize the incidence of the splenic infarction at postoperative 15 days, we considered to be late to do add pharmacotherapy. So we did not added any treatment for splenic infarction. Contrast-enhanced CT at 3 months postoperatively showed organization of the infarcted spleen (Fig. 3d). As of 1 year postoperatively, the patient has shown no particular complications.

## Discussion and conclusions

LSPDP is increasingly being applied for benign and low-malignant pancreatic tumors, thanks to significant progress in laparoscopic procedures. Although the patency of conserved splenic vessels and the rate of splenic infarction are important issues for LSPDP, few studies have reported on this problem.

Kimura et al. provided the first report of splenic vessel-conversing spleen-preserving distal pancreatectomy for a benign tumor [3]. To preserve the splenic artery and vein, careful ligation of the pancreatic tributaries was demanded, and the procedure is technically difficult and time-consuming. On the other hand, Warshaw’s method for the LSPDP procedure involves removal of the splenic artery and vein with the left pancreas, but meticulous preservation of the collateral blood supply from the short gastric and left gastroepiploic vessels.

Xinzhe et al. reported a meta-analysis of Warshaw’s methods found a significantly shorter operation time, but no difference between Warshaw’s method and Kimura’s method in the overall rate of complications, including the rate of pancreatic fistula. However, the frequencies of gastric varices and splenic infarction were significantly higher with Warshaw’s method [9]. Although no definitive consensus has been reached on which is the superior procedure, we prefer Kimura’s method due to the lower risk of splenic complications. Several reports have described splenic infarction after spleen-preserving distal pancreatectomy. Beane et al. reported 1 case of splenic infarction among a total of 45 cases, for a rate of 4.5% [10]. Worhunsky et al. reported 2 cases among a total of 13 cases (15.4%) [7], and Zhou et al. reported 33 cases among a total of 206 cases (16%) [6]. However, all of those involved partial infarction. We found a report stating that postoperative splenectomy was needed for splenic infarction after LSPDP using Kimura’s method [11]. That group reported three cases of re-operation and 1 case of postoperative splenectomy among 36 cases of LSPDP using Kimura’s procedure. We summarized recent studies about incident of splenic infarction after LSPDP in Table 1 [5–8, 10–12].

**Table 1:** Recent studies about incident of splenic infarction after laparoscopic distal pancreatectomy

Author	Year	Country	Reference No.	Study type	Technique	No. of patients	Splenic infarction (%)
Zhou ZQ	2014	China	6	R	Kimura	206	33 (16.0%)
					Warshaw	40	21 (52.5%)
Beane JD	2011	USA	10	R	Kimura	45	1 (2.2%)
					Warshaw	41	16 (39%)
Worhunsky	2014	USA	7	R	Kimura	13	2 (15.4%)
					Warshaw	15	7 (46.7%)
Butturini	2012	Italy	11	R	Kimura	36	1 (2.8%)
					Warshaw	7	1 (14.3%)
Hwang HK	2012	Korea	8	R	Kimura	29	0 (0%)
Yoon YS	2009	Korea	5	R	Kimura	22	4 (18.2%)
Lee LS	2016	Simgapore	13	R	Kimura	63	2 (3.2%)
					Warshaw	26	9 (34.6%)

*R* Retrospective, *Kimura* LSPDP with conserving splenic vessels, *Warshaw* LSPDP with division of splenic vessels

In the current case, we preserved the splenic artery and vein and the collateral blood supply from the short gastric and left gastroepiploic vessels. Even if obliteration of the splenic vessels had occurred, the splenic blood supply was expected to be maintained via the short gastric and left gastroepiploic vessels. However, splenic infarction still occurred. In our series of 10 LSPDPs, although another two cases showed obliteration of the splenic vessels, splenic infarction was not seen in either of those two cases. Two possible mechanisms were considered for the splenic infarction in the current case. The first was scattered micro-embolization to the spleen resulting from intraoperative procedures. Because the tumor was widely attached to the splenic vessels, we needed to draw on the tape encircling the splenic vessels to separate the tumor from the splenic vessels. Drawing on the tape strongly and for an extended period may have resulted in micro-emboli within the splenic artery flowing into the spleen, subsequently causing complete splenic infarction. The second mechanism might have involved the postoperative pancreatic fistula. Locally intense inflammation around the spleen might have contributed to splenic infarction, leading to obliteration of not only the splenic artery, but also the short gastric and left epigastric artery [13]. Overall, the first mechanism was considered more likely.

Conservation of splenic vessels in LSPDP is a demanding procedure. To prevent splenic infarction in LSPDP using Kimura’s method, the possibility of splenic infarction should be kept in mind and drawing tightly on the vessel loop encircling the splenic vessels should be avoided. If splenic infarction might have occurred intraoperatively, splenic arterial flow should be checked by intraoperative ultrasonography and splenectomy added if needed. Fortunately, the current splenic infarction did not require additive splenectomy after LSPDP.

To the best of our knowledge, this represents the first report of complete splenic infarction after LSPDP using Kimura’s method. Preventing splenic infarction in LSPDP requires careful isolation of the pancreatic parenchyma from the splenic vessels, and avoidance of drawing tightly on the vessel loop encircling the splenic vessels.

## Abbreviations

CT: Computed tomography
LSPDP: Laparoscopic spleen-preserving distal pancreatectomy
MRI: Magnetic resonance imaging
